# Experimental Analysis of Tear Fluid and Its Processing for the Diagnosis of Multiple Sclerosis

**DOI:** 10.3390/s23115251

**Published:** 2023-06-01

**Authors:** Vladimíra Tomečková, Soňa Tkáčiková, Ivan Talian, Gabriela Fabriciová, Andrej Hovan, Daria Kondrakhova, Katarína Zakutanská, Miriama Skirková, Vladimír Komanický, Natália Tomašovičová

**Affiliations:** 1 Department of Medical and Clinical Biochemistry, Faculty of Medicine, Pavol Jozef Šafárik University in Košice, Trieda SNP 1, 040 11 Košice, Slovakia; 2 Department of Medical and Clinical Biophysics, Faculty of Medicine, Pavol Jozef Šafárik University in Košice, Trieda SNP 1, 040 11 Košice, Slovakia; sona.tkacikova@upjs.sk (S.T.); ivan.talian@upjs.sk (I.T.); 3 Department of Biophysics, Institute of Physics, Faculty of Science, Pavol Jozef Šafárik University in Košice, Jesenná 5, 041 54 Košice, Slovakia; gabriela.fabriciova@upjs.sk (G.F.); andrej.hovan@upjs.sk (A.H.); 4 Department of Condensed Matter Physics, Institute of Physics, Faculty of Science, Pavol Jozef Šafárik University in Košice, Park Angelinum 9, 041 54 Košice, Slovakia; kondrakhova.darya@gmail.com (D.K.); vladimir.komanicky@upjs.sk (V.K.); 5 Department of Magnetism, Institute of Experimental Physics, Slovak Academy of Sciences, Watsonova 47, 040 01 Košice, Slovakia; zakutanska@saske.sk (K.Z.); nhudak@saske.sk (N.T.); 6 Department of Opthalmology, Faculty of Medicine, Pavol Jozef Šafárik University in Košice, Trieda SNP 1, 040 11 Košice, Slovakia; miriamaskirkova@gmail.com

**Keywords:** tear fluid, multiple sclerosis, Raman spectroscopy, infrared spectroscopy, proteomics, atomic force microscopy

## Abstract

A pilot analysis of the tear fluid of patients with multiple sclerosis (MS) collected by glass microcapillary was performed using various experimental methods: liquid chromatography–mass spectrometry, Raman spectroscopy, infrared spectroscopy, and atomic-force microscopy. Infrared spectroscopy found no significant difference between the tear fluid of MS patients and the control spectra; all three significant peaks were located at around the same positions. Raman analysis showed differences between the spectra of the tear fluid of MS patients and the spectra of healthy subjects, which indicated a decrease in tryptophan and phenylalanine content and changes in the relative contributions of the secondary structures of the polypeptide chains of tear proteins. Atomic-force microscopy exhibited a surface fern-shaped dendrite morphology of the tear fluid of patients with MS, with less roughness on both oriented silicon (100) and glass substrates compared to the tear fluid of control subjects. The results of liquid chromatography–mass spectrometry showed downregulation of glycosphingolipid metabolism, sphingolipid metabolism, and lipid metabolism. Proteomic analysis identified upregulated proteins in the tear fluid of patients with MS such as cystatine, phospholipid transfer protein, transcobalamin-1, immunoglobulin lambda variable 1–47, *lactoperoxidase*, and ferroptosis suppressor protein 1; and downregulated proteins such as haptoglobin, prosaposin, cytoskeletal keratin type I pre-mRNA-processing factor 17, neutrophil *gelatinase*-associated lipocalin, and *phospholipase A2*. This study showed that the tear proteome in patients with MS is modified and can reflect inflammation. Tear fluid is not a commonly used biological material in clinico-biochemical laboratories. Experimental proteomics has the potential to become a promising contemporary tool for personalized medicine, and it might be applied in clinical practice by providing a detailed analysis of the tear-fluid proteomic profile of patients with MS.

## 1. Introduction

Multiple sclerosis (MS) is an autoimmune-mediated, neurodegenerative disorder of the central nervous system (CNS) characterized by inflammatory demyelination with axonal transection. MS is the most common cause of non-traumatic neurological disability in young adults [1]. The progressive chronic inflammation leads to the formation of multiple foci of demyelinated lesions in the white and gray matter and finally to disability [2]. The diagnosis of MS and evidence of its dissemination in time and space are mostly based on clinical evaluation, neuroimaging by magnetic resonance imaging, and cerebrospinal fluid analysis, but no definitive diagnostic test exists [3,4].

MS symptoms vary according to the location of lesions occurring within the CNS [5,6]. The most common clinical presentations are unilateral optic neuritis, and brainstem and spinal cord syndromes [7]. Ocular symptoms may occur at any stage of the disease. Their course can be clinically latent or very intensive [8,9].

Tears lubricate the ocular surface, carrying secreted molecules from corneal epithelial cells [10]. Tears represent the whole physiological status of the body [11,12]. Quantitative determination of tear proteins by examining all three tear-fluid layers—lipid, aqueous, and mucous—is of increasing interest in ophthalmology [13]. In tear research and diagnostics, tear collection is a critical step. Precorneal tears, as a biological fluid, are easily accessible with non- or minimally invasive methods at a relatively low cost. The complexity of tear-fluid composition is a challenge for researchers [14]. The total protein concentration of human tears ranges from 6 to 11 mg/mL, with lysozyme being the most abundant tear protein with a concentration of approximately 1 mg/mL [15]. The main components of human tears are proteins, as well as tear lipids, metabolites, electrolytes, and vitamins [16].

The identification of protein biomarkers and molecular mechanisms of MS may be facilitated by proteomics as a promising tool for revealing molecular pathways and quantifying differentially expressed proteins [17,18]. Overall, proteomics, metabolomics, and bioinformatics approaches can help to develop different diagnostic methods for complex disorders such as MS, from biomarker discovery to personalized medicine [4].

Tears, as a non-invasive strategic material in the search for new protein biomarkers of the nervous system, seem to be an innovative tool for the early, non-invasive diagnosis of nervous system diseases [19]. HPLC proteomic experimental analyses and infrared (IR) and Raman spectral data analyses of tear fluid are shown in this paper. This pilot study analyzes the challenges of using different proteomic experimental analytical approaches on the complex tear fluid of patients with MS compared with control subjects.

## 2. Materials and Methods

### 2.1. Patients and Biological Material

In this pilot study, basal tear fluid from the lower eyelid of random patients with MS (*n* = 20) and healthy subjects (CTRL, *n* = 10) was collected by glass microcapillary (Drummond, Broomall, PA, USA) at the Department of Ophthalmology, University Hospital Louis Pasteur in Košice. Before measurement, the obtained samples of tear fluid were kept at −80 °C. Table 1 provides a thorough description of patients, procedures, and therapies. Milgamma and vitamins B and D were given to the patients as supplements, and they received care in accordance with the Helsinki Declaration. They signed the informed consent form after being made aware of the study’s goals and dangers. The revised 2017 McDonald criteria, which include the history, clinical manifestations, laboratory findings (cerebrospinal fluid-CFS), and data from neuroimaging, were used to make the diagnosis of MS. Many patients in this study presented the “relapsing–remitting” type of MS. Exacerbations and remissions are the hallmarks of this type of MS, which can progress into “secondary progressive” MS with growing disability in between episodes. Relapsing–remitting, primary progressive, and secondary progressive are the three main presentation patterns for MS, a chronic autoimmune disease of the central nervous system. The disability in “primary progressive” MS progresses right away.

The study received clearance from the Louis Pasteur University Hospital in Košice’s ethics committee (protocol code 2020/EK/06042 and date of approval 25 June 2020) for experiments utilizing human tear fluid.

### 2.2. Infrared Spectroscopy

Infrared spectra were acquired by FTIR spectroscope Vertex 80-v (Bruker, Mannheim, Germany). Experimental (*n* = 6) and control samples (*n* = 6) were prepared by the following procedure. Tear fluid (10 µL) obtained by glass capillary method was deposited on a ZnSe base and dried in a refrigerator. After drying a drop of the sample, another drop of tear fluid with a volume of 10 µL was deposited on top of the dry layer to obtain a thicker layer for the sample. After drying the second layer in a refrigerator, the infrared spectra were measured in transmission mode in a vacuum at ambient temperature.

### 2.3. Drop-Coating Deposition Raman Spectroscopy

In drop-coating deposition Raman (DCDR) measurements, 1.5 μL of the analyte (CTRL, *n* = 7; MS, *n* = 8) was dropped on a m-RIM^™^ plate (BioTools, Wobum, MA, USA) and left to dry at room temperature for about 40 min.

All spectra were obtained with a Raman confocal microspectrometer (Renishaw inVia, Gloucestershire, UK) equipped with a Leica direct microscope, an electrically cooled CCD camera, and 1800 lines/mm diffraction grating. Before the measurements, the system was calibrated and monitored by using a silicon reference (520.5 cm^−1^).

The laser beam was operated at the wavelength λ = 532 nm and 2 mW power to avoid heat-induced degradation of the sample. The beam was focused onto the specimen using a 50×/0.50 NA objective, which was used to collect the Raman signal in so-called backscattering geometry. An acquisition time of 10 × 20 s was used for each spectrum. For every sample, different spectra were measured at various locations in the dry sample to account for concentration gradients and inhomogeneities caused by protein segregation during water evaporation. Raman spectra were background-subtracted and normalized for clarity of presentation and better comparison. The background was subtracted using the rolling ball algorithm [20]. After the background subtraction, the data were normalized to the phenylalanine peak maximum at 1003 cm^−1^ and/or to the peak area of the amide group at 1680 cm^−1^. Comparable results were obtained by normalizing the spectra for both bands. Almost identical results were obtained using both bands for normalizing the spectra. The average of the normalized spectra of the control group was subtracted from each normalized spectrum of the tear sample to obtain the difference spectra.

### 2.4. Atomic-Force Microscopy

Atomic-force microscopy (AFM) is a technique that allows obtaining high-resolution images of any surface by using a sharp-tip scanning probe. AFM is widely used for measurements of the morphology and roughness of various biomaterials [21,22]. AFM is an ideal tool in interdisciplinary research to detect and study pathological conditions even in the earliest stages.

In this study, tear fluid (5 µL) of patients with MS (*n* = 5) and healthy subjects (*n* = 5) was analyzed by AFM analysis using an atomic-force microscope (ICON, Bruker, Billerica, MA, USA) in tapping mode with silicon tips (MicroMasch, Berkley, CA, USA, NSC35 series) and the radius of curvature of 10 nm on two different substrates: microscopic glass (with a size of 3 × 8 cm) and oriented silicon (100) substrate (with a size of 1 × 1 cm). Tear fluid (5 µL) was dripped on the substrate without smearing, and it was dried at room temperature without fixative [23]. Data were processed using a nanoscope ScanAsyst™ software 8.15 (Bruker, Billerica, MA, USA).

### 2.5. HPLC Mass Spectrometry

Tear fluid (15 µL) was diluted with 15 µL of 8 M urea in 0.05 M ammonium bicarbonate. Reduction of disulfides to dithiols in tear-fluid proteins was performed with the addition of 5 mM final concentration of dithiothreitol (BioRad, Hercules, CA, USA) and incubation for 30 min at 37 °C. The alkylation by using 14 mM final concentration of iodoacetamide (BioRad, Hercules, CA, USA), for 30 min at 37 °C, took place in the dark. The tear-fluid proteins were reduced and alkylated to break disulfide bridges and ‘cap’ the reduced cysteines. The urea concentration was lowered to 0.8 M by adding 0.001 M CaCl_2_ (Sigma, St. Louis, MO, USA). In the next step, tear-fluid proteins were digested using 0.5 µg trypsin (Promega, Madison, WI, USA) per sample, overnight at 37 °C. The digestion with trypsin was stopped by the addition of 20% formic acid (Merck, Darmstadt, Germany) to obtain pH ≤ 3. Acetonitrile 3 µL (Merck, Darmstadt, Germany) was added to the tear fluid and centrifuged at 14,000× *g*, 4 °C, for 20 min. The supernatant (2 µL) was injected into a nano HPLC system, the Ultimate 3000 RSLC NCP (Thermo Scientific, Waltham, MA, USA), coupled with an AmaZon speed ETD ion trap mass spectrometer (Bruker Daltonik, Billerica, MA, USA) for proteome profiling. Peptides were preconcentrated on the trap column Acclaim PepMap 100 (Dionex, Thermo Scientific, CA, USA), 100 µm × 2 cm, C18, 5 µm particles with water–acetonitrile loading solvent in the ratio 2:98 *v*/*v* containing 0.1% formic acid at flow 8 µL·min^−1^. The peptides were then eluted and separated on a homemade capillary column 75 µm × 30 cm, packed with reversed phase C18, 3 µm particles (Magic C18 AQ, Michrom Bioresources, Auburn, CA, USA). Mobile phases consisted of 0.1% formic acid in a water–acetonitrile mixture in the ratio 98/2 *v*/*v* (A) and 0.1% formic acid in the water–acetonitrile mixture in the ratio 5/95 *v*/*v* (B) operated at a constant flow rate of 0.3 µL per minute. The samples were measured in auto MS/MS mode with 10 precursors for 1 MS scan; only 2+ and 3+ precursors were taken for fragmentation with an active exclusion set to 0.5 min. The ICC target was set to 400,000 for MS and MS/MS scans, and the maximum accumulation time was 0.050 s for MS and 0.1 s for MS/MS. The isolation window was set to 2.2 Da, and the scan range was 300–1300 Da.

The Mascot 2.4 (MatrixScience, London, UK) search engine against the Swiss-Prot database was used to identify proteins. The parameters were determined as follows: taxonomy—Homo sapiens (human), variable modification: oxidation of methionine, fixed modification: carbamidomethylation of cysteine; MS tolerance: 0.6 Da; MS/MS tolerance: 0.6 Da; and the false discovery rate (FDR) threshold was set to 1%.

The pathway enrichment analysis (PEA) was performed by the Reactome Analysis Service (https://reactome.org, accessed on 10 May 2023) from the comparison of 5 patients with MS versus 5 controls. Each sample was measured twice, and at the end, 10 versus 10 measurements were analyzed.

## 3. Results

### 3.1. Infrared Spectroscopy

Infrared spectroscopy is a suitable method for exploring protein structure. The infrared spectra of proteins have nine characteristic peaks: Amide I–Amide VII, Amide A, and Amide B. The most significant and least complex peaks are Amide I (located at 1600–1700 cm^−1^), which corresponds to C-O stretching; Amide II (located at 1480–1575 cm^−1^), which corresponds to N-H bending and C-N stretching; and Amide A (located at about 3300 cm^−1^), which corresponds to N-H stretching [24].

**Figure 1:**
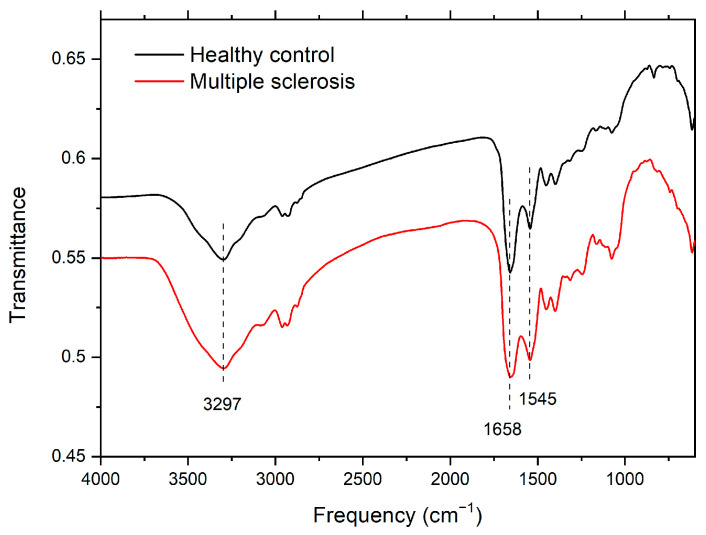
Representative spectra of tear fluid from healthy subjects and patients with MS. Denoted values correspond to peaks Amide A, Amide I, and Amide II, from left to right, respectively.

Representative infrared spectra of healthy controls and patients with MS are shown in Figure 1. No significant differences were found between the healthy control and MS spectra. In both spectra, all three significant peaks are present, and they are located at around the same positions.

**Figure 2:**
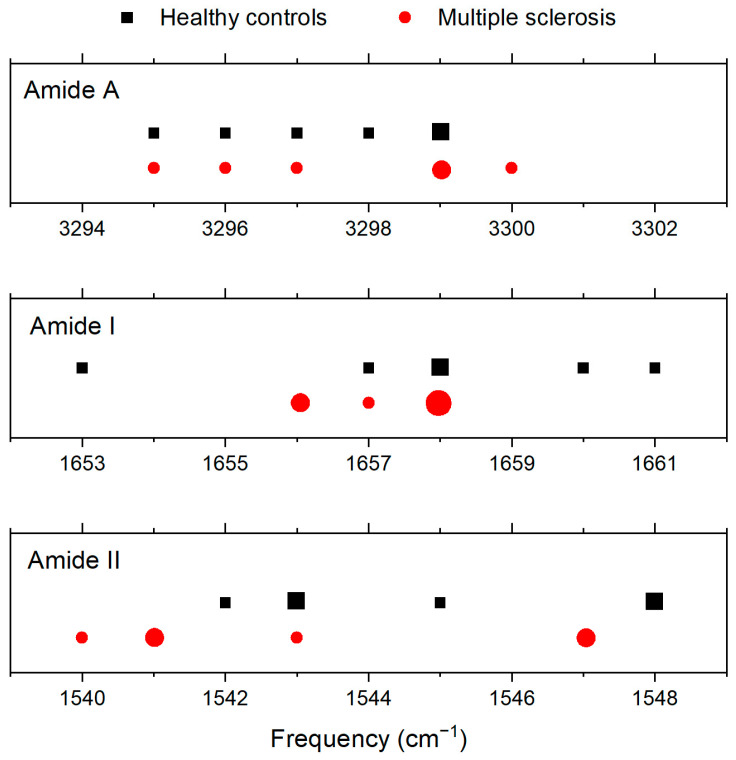
Positions of peaks Amide A, Amide I, and Amide II for healthy controls and for patients with MS. The size of the symbol reflects the number of spectra with a given peak position. Frequency intervals for MS overlap with intervals for healthy controls (see the x-axis). No quantity is plotted on the y-axis, and data points for MS were plotted under data points for healthy controls for clarity.

The positions of the Amide A, Amide I, and Amide II peaks are listed in Table 2. The average values for healthy controls (*n* = 6) and for patients with MS (*n* = 6) differed by at most 2 cm^−1^, and the intervals in which the peaks are located for MS overlap the intervals for the healthy controls (see Figure 2). Infrared spectroscopy suggests that there are no changes in the proteins visible by infrared spectroscopy in the case of MS. There being no changes in the measured infrared spectra of patients with MS can be caused by the small concentrations of proteins affected by MS, whose signal is dominated by the signal of more concentrated proteins.

### 3.2. Drop-Coating Deposition Raman (DCDR) Spectroscopy

For Raman analysis, each tear-fluid sample was dried on the hydrophobic surface to form the coffee-ring pattern to concentrate the weak protein solution. The Raman signal of the hydrophobic surface has a negligible effect on the Raman spectra of tear fluid.

**Figure 3:**
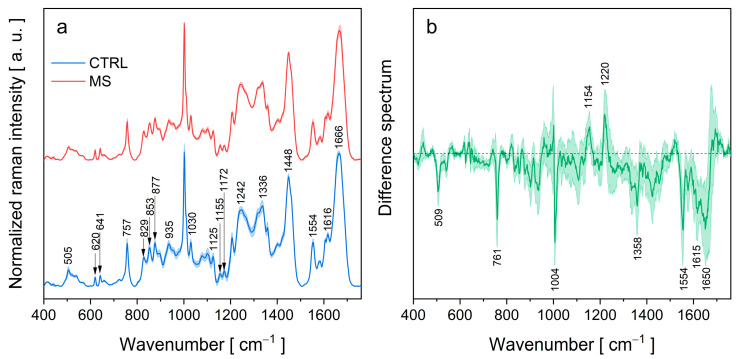
Average Raman spectra obtained from tears of patients with MS and CTRL: (**a**) The difference between the spectrum for MS and for average CTRL; (**b**) the shadowed area refers to the standard deviation of the data.

Figure 3a shows the normalized Raman spectra of tears in the range 400–1750 cm^−1^ for patients with MS and healthy controls. The solid line represents the average Raman spectrum, and the shaded area represents a standard error. The spectra of both groups (MS and CTRL) have the same position of peaks.

The most intense bands in the spectra are primarily associated with the vibrations of amide bonds and functional groups of aromatic amino acids. Well-defined bands in the spectra correspond to vibrations of the phenylalanine ring (620, 1002, and 1616 cm^−1^), tryptophan ring (757, 877, 1125, doublet 1336/1358, and 1554 cm^−1^), tyrosine ring (641, doublet 829/853, 1155, 1172, and 1616 cm^−1^), and amide group (1242, 1263, and 1666 cm^−1^) [25,26,27]. The positions of the amide bands reflect the secondary structure of the protein. Typical wavenumbers for α-helix and β-sheet structures are 1655–1662 and 1264–1272 cm^−1^ (α) 1672–1676 and 1227–1242 cm^−1^ (β) for Amide I and Amide III modes, respectively [25]. This is consistent with the fact that tear fluid contains proteins possessing α-helix and/or β-sheet structures, such as lysozyme, lactoferrin, and lipocalin. A broad peak near 1448 cm^−1^ originates from the deformation motion of CH_2_ and CH_3_ groups, and a band around 935 cm^−1^ corresponds to a backbone N−C_α_−C stretching vibration of α-helix secondary structures [28]. In the Raman spectrum of tears, we also observe less intense bands belonging to S−S stretching (505, 520, and 539 cm^−1^) (Table 3). 

The average difference spectra of the MS and control tear-fluid samples are shown in Figure 3b. The minima occurring at 761, 1004, 1358, 1554, and 1615 cm^−1^ indicate a decrease in tryptophan and phenylalanine content in the tear fluid of MS patients (Table 3).

Another minimum in the difference spectrum is observed at 509 cm^−1^ (Figure 3b), which is probably caused by a decrease in the content of proteins containing disulphide bridges. In addition, the minimum at 1650 cm^−1^ and the relative maximum at 1220 cm^−1^ in the difference spectrum (Figure 3b) indicate that in the tear fluid of the MS patients, there is a change in the relative contribution of secondary structures of polypeptide chains, a decrease in the α-helix and an increase in the β-sheet structure.

### 3.3. Atomic-Force Microscopy

**Figure 4:**
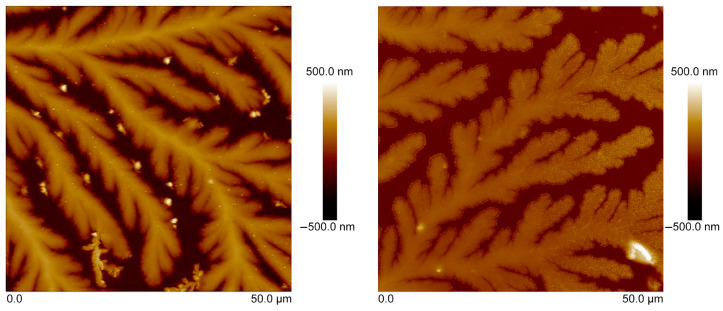
AFM topography of tear fluid of CTRL (**left**) and MS (**right**) studied on an oriented silicon (100) substrate.

**Figure 5:**
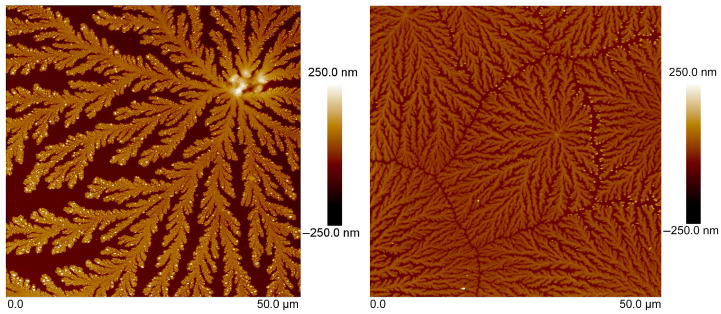
AFM topography of tear fluid from CTRL (**left**) and MS (**right**) studied on a microscopic glass slide.

The surface morphology of tear fluid in the control samples (CTRL) obtained from healthy subjects as well as the samples of tear fluid with MS was studied on two different substrates (oriented silicon (100) substrate and microscopic glass slide) by the AFM method (Figure 4 and Figure 5).

Note that analysis of tear-fluid samples (CTRL and MS) on two types of substrates showed similar structures with similar types of crystal growth and symmetry. The differences in the topography of the samples obtained on different types of substrates are minimal. The roughness of the tear fluid on the silicon substrate was higher than on the glass microscope slide. Tear fluid from patients with MS showed lower roughness on both substrates compared to CTRL tear fluid. Surface morphology shows the formation of fern-shaped dendrites that differed in lengths of branches, thickness, and surface roughness between the CTRL and MS tear-fluid samples.

High surface roughness, above Ra = 300 nm, was observed for CTRL tear-fluid samples studied on an oriented silicon substrate compared to the finer structure of MS tear fluid, which had a surface roughness of about Ra = 70 nm. Crystals with an average height of about 200 nm, and dendrites with an average branch width of 10 µm and a length of about 50–60 µm, were observed on the surface of tear fluid with MS (Figure 4). The average length of dendrites of main branches in the CTRL tear fluid was more than 80 µm, and the width was about 10–15 µm. The roughness of the surface was Ra = 95 nm. The height of individual crystals reached 300 nm.

The surface roughness of CTRL tear fluid studied on a glass microscopic slide was about Ra = 59 nm, while finer MS tear fluid showed a lower Ra = 25 nm surface roughness, a smaller average crystal height (above 100 nm), and an average branch length of dendrites (about 25 µm) with 3–4 nm width (Figure 5). The average 50 µm length of the main branches of CTRL tear fluid studied on a microscopic glass slide was longer and thicker, with a width of about 10 µm and separate crystals with a height of up to 200 nm compared to MS tear fluid.

### 3.4. HPLC Mass Spectrometry—Bottom Up Proteomic Analysis

**Figure 6:**
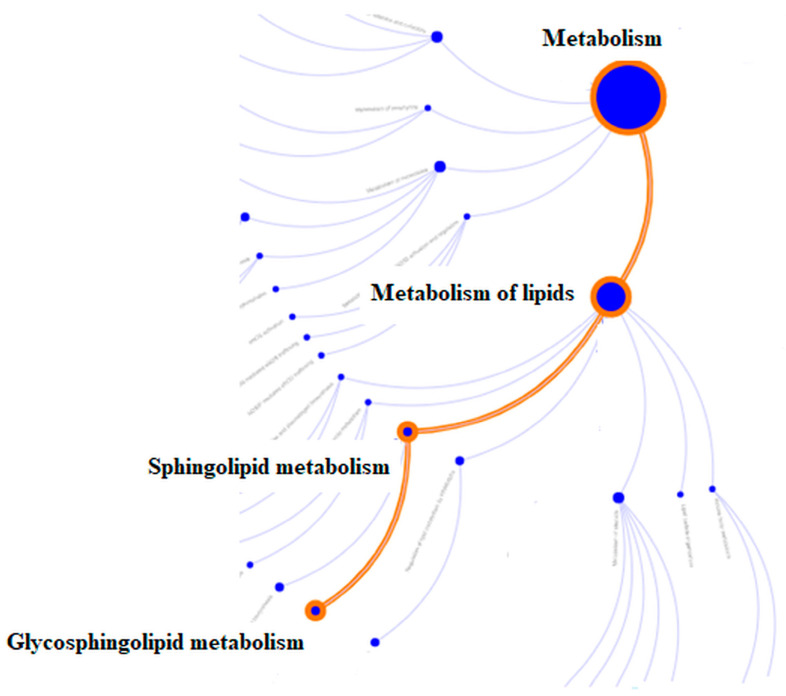
The simple scheme of lipid, sphingolipid, and glycolipid metabolism.

These results summarize the pathway reactome analysis created by the Reactome Analysis Service (https://reactome.org, accessed on 10 May 2023). Our results from pathway enrichment analysis (PEA) demonstrated that the metabolism of lipids (sphingolipid and glycosphingolipid pathways) was downregulated (Figure 6, Table 4). All dysregulated MS tear-fluid proteins (Table 5 and Table 6) were identified by weighted gene-set analysis.

Downregulated proteins (Table 5) from PEA and upregulated proteins (Table 6) in the tear fluid of patients with MS were identified by proteomic analysis. The top downregulated proteins (Table 5) were haptoglobin (HPT_HUMAN), prosaposin (SAP_HUMAN), cytoskeletal keratin type I (K1C13_HUMAN), pre-mRNA-processing factor 17 (PRP17_HUMAN), neutrophil *gelatinase*-associated lipocalin, and *phospholipase A2* (NGAL_HUMAN).

The top upregulated proteins (Table 6) were cystatin C (CYTC_HUMAN), phospholipid transfer protein (PLTP_HUMAN), transcobalamin-1 (TCO1_HUMAN), immunoglobulin lambda variable 1–47 (LV147_HUMAN), *lactoperoxidase* (PERL_HUMAN), and ferroptosis suppressor protein 1 (FSP1_HUMAN) (Table 3).

## 4. Discussion

Different biological samples from MS patients, including blood, cerebrospinal fluid, and CNS tissue, have been subjected to proteomic analysis. Blood, tissue, and cerebrospinal fluid are the samples that must be collected through invasive procedures. Sweat, tears, saliva, and urine are the non-invasive samples [19]. The CNS and tears are linked. It is a fascinating pure body liquid, free of any pigment that may interfere with spectral analysis. It was discovered that tears contained the same proteins found in cerebrospinal fluid. Tear fluid is a body fluid with a composition like cerebrospinal fluid and blood [19]. More than 1300 proteins were discovered in the tear fluid of healthy individuals [19]. The major proteins found in tears are lysozyme, lactoferrin, tear-specific prealbumin, and secretory immunoglobulin A. Lysozyme has the highest concentration of 0.5–4.5 mg/mL [31], and it represents about 40% of the protein content in tears [32]. The concentration of lactoferrin is 1–2 mg/mL (about 25% of total protein content) [31], and the concentrations of tear-specific prealbumin and secretory immunoglobulin A are 0.5–1.5 mg/mL (10–20%) [31] and 71 μg/mL–2.4 mg/mL [33], respectively. Of all biomarkers of MS, only alpha-1 antichymotrypsin demonstrated a notable rise among promising proteins, including proline-rich protein 4 and protein 16 homolog B [3]. Immunoglobulin G (IgG) was studied in tear fluid in connection with MS [34]. The infrared signal from proteins affected by MS may be dominated by major proteins, which result in identical spectra for healthy subjects and patients with MS. Upregulated immunoglobulins (antibodies) were detected by HPLC MS spectroscopy. The immunoglobulin light chain’s variable domain’s V region plays a role in antigen recognition [35]. When an individual antigen binds to the membrane-bound immunoglobulins during the recognition phase of humoral immunity, these receptors cause the B lymphocytes to develop and differentiate into immunoglobulin-secreting plasma cells [36]. The effector phase of humoral immunity is mediated by secreted immunoglobulins, which remove bound antigens. The V-(D)-J rearrangement assembles the variable domains, which increases the possibility of somatic hypermutations, allowing selective affinity for a specific antigen [37]. Pre-mRNA-processing factor 17 is required for pre-mRNA splicing. Differences in aberrant alternative pre-mRNA splicing are related to the genetic risk variations for MS [38,39].

Oligoclonal IgG bands are a characteristic sign of inflammation in MS patients’ cerebrospinal fluid and tear fluid. They can be used as a diagnostic tool for individuals who may be experiencing their first suspected demyelinating episode [34].

Infrared spectroscopy is not a suitable method for monitoring changes in protein structure in patients with MS in contrast to other diseases such as diabetes mellitus [23], eye diseases [40], or depressive disorders [41], as we showed previously. Other experimental techniques, such as Raman spectroscopy, atomic-force microscopy, and especially liquid chromatography–mass spectrometry, are more suitable for MS.

Raman scattering is extremely responsive to changes in the secondary structure of proteins and variations in the amount of amino acid residues and their microenvironment, particularly those with aromatic functional groups [25]. As a result, our Raman findings point to a problem with tryptophan metabolism. This may affect how neurotransmitters are regulated, leading to serious neurological dysfunction [42].

Using NMR metabolomic analysis, different metabolic profiles were distinguished in MS patients as compared to the CTRL group [43]. The main metabolic changes were associated with tryptophan metabolism and energy metabolism. Tryptophan levels in the serum of MS patients were shown to be declining. Additionally, tryptophan levels in the cerebral fluid were shown to be lower in people with secondary progressive MS [44].

It has been shown that dysregulation of haptoglobin (Hpt) in cerebrospinal fluid can reflect neurological diseases [5]. It was suggested that MS research be conducted on the tears collected from MS patients using glass microcapillaries to avoid cerebrospinal fluid analysis, which requires an invasive collection [3].

Our proteomic research found that the Hpt proteins were upregulated in the tear fluid of MS patients. Hpt modulates the acute phase response. Hpt functions as an antioxidant and has antibacterial properties. Under stressful circumstances, hemolysis of erythrocytes causes a buildup of hemoglobin (Hb) in the kidneys, which is then released in the urine. For hepatic recycling of heme iron and avoiding kidney injury, Hpt catches and mixes with free plasma Hb. Through an endocytic lysosomal breakdown route, Hb/Hpt complexes are quickly eliminated by the macrophage CD163 scavenger receptor expressed on the surface of liver Kupfer cells. In MS, high levels of inflammation are characterized by a rise in IL-6, which in turn affects how Hpt is expressed. This illness also has a connection to inflammation, and it progresses with muscle breakdown, which has a connection to hemolysis. Due to the free (Hb) pro-oxidative effect, hemolysis accelerates muscle destruction. A plasmatic Hpt rise, on the other hand, encourages its union with free Hb (Hpt-Hb). Pro-oxidative action appears in tear fluid due to the declining Hpt levels. A potential indicator of muscle recovery is the involvement of Hpt, whose expression is dependent on the synthesis of IL-6, in the pathogenesis of MS. In patients with MS who are obese or have skeletal muscle degeneration, the blood levels of Hpt are elevated. Due to the increased fragility of erythrocytes in MS patients, Hpt serves as the first line of defense for myelin against elevated Hb levels that are released. MS affects the basic myelin proteins and changes the blood–brain barrier [45].

A risk factor for MS could be elevated cystatin C levels, which were discovered to be an independent predictor of cognitive impairment. It is a possible sign of the diagnosis of MS in the cerebrospinal fluid and tear fluid [46]. Amyloid-beta binding and cysteine proteinases are both inhibited by statin C. In many inflammatory illnesses, excessive neutrophil degranulation is a common characteristic. Since they are the most prevalent circulating and first-responding innate myeloid cells, neutrophils have received too little attention in the setting of multiple sclerosis (MS). Neutrophils are responsible for the release of inflammatory mediators and enzymes like interleukin-1, myeloperoxidase, and different proteinases, the phagocytosis of myelin (as debris), the release of neutrophil extracellular traps, the production of reactive oxygen species, the breakdown of the blood–brain barrier, and the production and presentation of autoantigens. Numerous neuroinflammatory brain disorders, including neurodegeneration, share the common mechanism of neutrophil-driven inflammation [47,48].

Innate immune reactions depend on active leukocytes releasing *myeloperoxidase*. Myeloperoxidase generates hypochlorous acid (HOCl) and other strong oxidants, which destroy bacteria and other invasive pathogens. *Myeloperoxidase* promotes chronic inflammatory diseases [49]. *Myeloperoxidase* is a heme-containing *oxidoreductase* that contributes to immune defense. On inflamed mucous surfaces, recruited neutrophils and eosinophils, respectively, release heme *peroxidases* [50]. *Lactoperoxidase* gene deletion results in tumors and multisystem inflammation in mice [51]. Adaptive and innate immune responses, as well as the cell death of oligodendrocytes and neurons and tissue damage due to oxidative stress, were seen at different phases of MS [52]. The release of iron from the myelin sheath during demyelination, mitochondrial dysfunction along with subsequent energy failure, impaired oxidative phosphorylation, inflammation, and the production of free radicals by activated immune cells are examples of impaired redox signaling [53].

Lipid peroxidation caused by an excess of ROS and iron and a deficiency of antioxidant glutathione (GSH) and glutathione peroxidase (GPX) levels leads to ferroptosis. Dysregulated ferroptosis contributes to neurodegeneration, tissue damage, inflammation, and infection. ROS, iron, and lipids induce ferroptosis. During ferroptosis, the cell membrane does not burst but forms blisters. In addition to the decreased or missing mitochondrial crests, the outer mitochondrial membrane rips. Upregulation of ferroptosis suppressor protein 1 inhibits ferroptosis [6]. The mechanisms causing autoimmune disorders involve the cell-death modes of ferroptosis and pyroptosis [54].

An iron-trafficking protein known as neutrophil *gelatinase*-associated lipocalin binds to iron, provides iron to the cell, or removes iron from the cell in innate immunity, ferroptosis, and other processes. The iron-loaded form raises intracellular iron concentration without causing apoptosis. The iron-free form decreases intracellular iron levels and induces the production of the proapoptotic protein BCL2L11/BIM and apoptosis because of interleukin-3 (IL3) deficiency. Since neutrophil *gelatinase*-associated lipocalin causes neutrophil infiltration, migration, and activation and can increase the T-helper 17 response, it influences neutrophil function. Investigations have shown that neutrophil *gelatinase*-associated lipocalin expression and secretion can be induced by IL-17 signaling pathways alone or combined with tumor necrosis factor (TNF)-α [55].

Neutrophil *gelatinase*-associated lipocalin is a protein that can bind tiny hydrophobic compounds. It is a diagnostic indicator of intrinsic organ injury, inflammation, and damaged cell necrosis [56]. Neutrophil *gelatinase*-associated lipocalin affects both neuroinflammation and neuroprotection. In response to inflammatory conditions, astrocytes in the brain produce and exude the majority of neutrophil *gelatinase*-associated lipocalin. Sequestered neutrophil *gelatinase*-associated lipocalin causes the astrocytes and microglia to become reactive and amplify the response. In pathological conditions, iron accumulation and a ferric/ferrous ion imbalance are the causes of apoptosis, necrosis, autophagy, and ferroptosis. In fact, the cortex and hippocampus of people with neurodegenerative disorders, such as MS, Alzheimer’s disease, and others, exhibit iron buildup [57].

A special class of lysosphingolipid called sphingosylphosphorylcholine has been researched in cardiovascular, neurological, and inflammatory diseases. Sphingosylphosphorylcholine controls how the keratin network architecture is structured. The keratin rearrangement brought on by phosphorylation accounts for the lower mechanical resilience. The intermediate filament proteins known as keratins control cytoarchitecture, epithelial-cell structural integrity, cell development, apoptosis, and cell motility in healthy adult tissues as well as in certain disorders like MS. Inflammation changes cell migration and motility. A bioactive lipid called sphingosylphosphorylcholine causes the cytoplasm to relax, keratin filaments to collapse within the nucleus, phosphorylation, and cell migration. JNK and ERK, two members of the mitogen-activated protein kinase family, have recently been demonstrated to facilitate keratin’s perinuclear rearrangement during inflammation [58].

In neurodegenerative diseases like MS, altered lipid metabolism and *phospholipase A2* deregulation are the main causes of inflammation. The enzymes known as *phospholipases A2* break down the fatty acids in the phospholipids of the membrane. Products include polyunsaturated fatty acids like arachidonic acid and lysophosphocholin. Pro-inflammatory thromboxanes and leukotrienes are produced by arachidonic acid via *cyclooxygenases* and *lipoxygenases*, promoting reactive oxygen species formation. Myelin loss and nerve-tissue deterioration have been linked to excessive ROS production. Erythrocytes typically have larger diameters, altered molecular profiles and lipid metabolisms, all of which are positively connected with the development of MS. In MS patients, prostaglandins, leukotrienes, ceramides, and sphingosine levels are elevated. Ceramide promotes the accumulation of oxygen species in hippocampus glial cells, which results in neuronal death and is involved in developing neurodegenerative disorders. *Phospholipase A2* activation and arachidonic acid release are linked to TNF-mediated signaling, with the latter leading to the activation of *sphingomyelinases* that hydrolyze sphingomyelin to create ceramide. This mechanism underpins the cytokine imbalance, which in turn alters the metabolism of phospholipids and sphingolipids [59]. Together with glycerophospholipids, sphingolipids are essential for numerous cellular processes, such as cell division, signaling processes, apoptosis, and engagement in pro- or anti-proliferative pathways. Sphingolipids are involved in the etiology of MS and are highly expressed in the central nervous system [60].

People with MS have dysregulated lipid metabolism. Regulated lipid metabolism is important for the remyelination process [61]. Elevated phospholipid transfer activity may be the cause of the reduced lipid uptake and aberrant myelin lipids seen in MS patients [62,63]. Human tears contain phospholipid transfer proteins. Phospholipid transfer protein can scavenge and transport phospholipids straight to the tear fluid’s surface layer. Instead of phospholipid transfer protein, tear lipocalin is where most phospholipids in tears are bonded. The phospholipid on the ocular surface can be bound and removed by tear lipocalin [64]. Phospholipid transfer protein mediates the exchange of phospholipids between triglyceride-rich lipoproteins, as well as the transfer of phospholipids and free cholesterol from triglyceride-rich lipoproteins (low-density lipoproteins, or LDLs, and very low-density lipoproteins, or VLDLs) into high-density lipoproteins (HDLs). Phospholipid transfer protein plays a significant role in HDL remodeling by facilitating the transfer of a variety of different lipid molecules, including diacylglycerol, phosphatidic acid, sphingomyelin, phosphatidylcholine, phosphatidylinositol, phosphatidylglycerol, cerebroside, and phosphatidylethanolamine.

Prosaposin is a lysosomal protein that controls inflammation by activating *hydrolases* involved in glycosphingolipid metabolism in the lysosome [65]. All mammalian saposins are created from a single prosaposin precursor molecule. Saposins, often referred to as SAPs (sphingolipid activator proteins), are small, non-enzymatic, low-molecular-weight lysosomal proteins that activate *hydrolases*, which are specialized lysosomal enzymes involved in lipid and sphingolipid breakdown [66]. The lack of saposin B causes neurologic deficits such as demyelination, periventricular white-matter abnormalities, and peripheral neuropathy [67]. Overactivation of platelet–neutrophil pairs was seen in MS-related inflammation and its effects on immunological responses [68]. The metabolism of cells influences their inflammatory characteristics. Prosaposin links mTOR signaling and the expression of macrophages [69]. Macrophages have two functions in the pathogenesis of MS. By producing inflammatory mediators involved in developing demyelination in MS, they can have neuroprotective and growth-promoting effects but can also cause tissue damage [70,71]. Numerous neurodegenerative illnesses’ pathogenesis is greatly influenced by chronic inflammation. When a triggering insult no longer exists, the acute inflammatory response ends, preventing a chronic inflammatory state in physiological conditions. In patients with various chronic inflammatory disorders, including MS, a neurodegenerative condition marked by persistent inflammation, several pathways control the resolution of inflammation and pro-inflammatory NF-kB signaling [72]. Inflamed and infected tissues quickly draw neutrophils from the bloodstream, where they quickly attract other inflammatory cells by releasing pro-inflammatory chemokines and cytokines. This process is essential for innate immunity. Inflammatory conditions can result from overrecruitment. In many acute and chronic inflammation cases, such as encephalomyelitis-MS, platelets are necessary for neutrophil recruitment [73].

The primary component of secondary granules in neutrophils during the neutrophil degranulation pathway is transcobalamin. Cofactors in circulation are cobalamins. Vitamin B12 binds to transcobalamin. Acquired hyperhomocysteinemia is most frequently caused by vitamin B12 and folate insufficiency. Cobalamin, generally known as vitamin B12, is essential for the structural and functional integrity of the nervous system. A lack of cobalamin causes demyelination, axonal degeneration, and ultimately irreversible damage from axonal death. Therefore, a B12 shortage might cause some clinical characteristics like those of MS patients. The severity of the disability and length of the illness were not correlated with vitamin B12 insufficiency [74].

## 5. Conclusions

According to this study, MS patients have altered tear proteomes. The tear proteome may be a good indicator of MS-related disorders and CNS inflammation. Tear fluid is not a biological substance that is frequently employed in clinico-biochemical laboratories. The vast diagnostic potential of such a small volume is currently only being explored experimentally. In this research, specific experimental and computer-solved techniques for MS patients’ tear fluid are described. In conclusion, it is known that a variety of conditions can raise the probability that MS will develop and progress, but the exact cause of this illness is still mostly unknown. The development of MS is closely associated with stress and disruption of lipid metabolism, as is common for other inflammatory neurodegenerative disorders. An autoimmune inflammatory response directed largely against the oligodendrocytes in the CNS triggers the onset of MS and results in demyelination and inflammation. After a period, when the oligodendrocytes’ potential for regeneration is depleted, the inflammatory processes affect the neurons themselves, causing neurodegeneration and lasting damage to the CNS [75]. By offering a thorough investigation of the proteome profile of MS patients, experimental proteomics has the potential to be used in clinical practice for the diagnosis of MS. Finally, clinical proteomic analysis techniques may develop into a modern tool for personalized therapy.

## Figures and Tables

**Table 1:** Description of healthy subjects (CTRL) and patients with MS.

Average	CTRL	MS
Patients	10	20
Females/Males	10/0	18/2
Age (years)	26.8 ± 3.02	33.3 ± 5.3
Retinal nerve fiber layer (μm)	-	101.4 ± 15.8
Ganglion cell complex (μm)	-	90.5 ± 11.9
Medication	0	20
No medication	10	-
REBIF (interferon β-1a)	-	10
AVONEX (interferon β-1a)	-	7
TECFIDERA (dimethyl fumarate)	-	1
OCREVUS (ocrelizumab)	-	2

**Table 2:** Positions of peaks Amide A, Amide I, and Amide II for CTRL and for patients with MS.

	Amide A	Amide I	Amide II
	Frequency (cm^−1^)	Frequency (cm^−1^)	Frequency (cm^−1^)
CTRL	3299	1658	1543
	3295	1653	1543
	3298	1657	1542
	3297	1658	1545
	3296	1660	1548
	3295	1661	1548
Average	3297	1658	1545
MS	3295	1658	1547
	3295	1658	1547
	3300	1658	1541
	3297	1656	1543
	3296	1656	1541
	3299	1657	1540
Average	3297	1657	1543

**Table 3:** Peak positions and tentative assignments of the Raman bands of human tears.

Peak Position (cm^−1^)	Assignment	Ref.
1666	Amid I: stretching C=O	[25,28,29]
1616	Trp, Phe, Tyr: n_8a_, ring stretching	[25,29,30]
1554	Trp: stretching C_2_=C_3_, W3 mode	[25,29]
1448	bending CH_2_	[25,28]
1358	Trp: Fermi resonance between in-plane N−C stretching and combination bands of ring out-of-plane deformations, W7 mode	[27,29]
1336	Trp, C−C_α_−H bending, C_α_−C stretching	[25,29]
1317 sh	aliphatic sidechain stretching vibrations	[25]
1263 sh	amid III (α)	[25,29]
1242	amid III (β)	[25,29]
1206	Tyr: n_7a_, C−CH_2_, Phe	[28,29]
1172	Tyr: n_9a_, CH in-plane bending	[29,30]
1100/1125	stretching C−N	[25]
1030	Phe	[25]
1002	Phe	[25]
954	Trp, Val	[25]
935	stretching N−C_α_−C (α)	[25,28,29]
877	Trp: benzene ring breathing and deformation N−H, W17 mode	[28]
853/829	Tyr doublet: Fermi resonance between ring breathing mode and overtone of out-of-plane ring bending mode	[25,28,29]
757	Trp: W18 mode	[25,29]
641	Tyr: n_6b_, ring deformation	[25,30]
620	Phe: in-plane ring deformation	[28]
539	S-S stretching (*trans-gauche-trans*)	[25]
520	S-S stretching (*gauche-gauche-trans*)	[25,29]
505	S-S stretching (*gauche-gauche-gauche*)	[25,29]

**Table 4:** Top regulated pathways including the corresponding false discovery rate (FDR), average fold change (Av.FC), and the number of identified genes (N Genes) obtained by Reactome Analysis.

Identifier	Regulation	FDR	Av.FC	N Genes
R-HSA-1660662 Glycosphingolipid metabolism	down	0.033	−1.662	1
R-HSA-428157 Sphingolipid metabolism	down	0.033	−1.662	1
R-HSA-556833 Lipid metabolism	down	0.044	−1.086	3

**Table 5:** List of top downregulated proteins with corresponding values of the logarithm of fold change (logFC), *p* value (*p*.Value), and adjusted *p* value (adj.P.Val).

Identifier	logFC	*p*.Value	adj.P.Val
PIP_ HUMAN	−0.5873613	0.0000270	0.0014060
SAP_HUMAN	−1.6620236	0.0016957	0.0440891
PIGR_HUMAN	−0.3813822	0.0045466	0.0788082
HPT_HUMAN	−1.8960652	0.0067941	0.0883235
PRP17_HUMAN	−1.0899125	0.0132627	0.1379316
K2C1B_HUMAN	−0.6535084	0.0281095	0.1995068
K1C13_HUMAN	−1.1428253	0.0276666	0.1995068
NGAL_HUMAN	−0.9558929	0.0383667	0.1995068
PA2GA_HUMAN	−0.8169733	0.0380019	0.1995068
CLUS_HUMAN	−0.4132970	0.0503125	0.2378411

**Table 6:** List of top upregulated proteins with corresponding values of the logarithm of fold change (logFC), *p* value (*p*.Value), and adjusted *p* value (adj.P.Val).

Identifier	logFC	*p*.Value	adj.P.Val
TCO1_HUMAN	0.6126178	0.0373045	0.1995068
CYTC_HUMAN	1.0627939	0.0658102	0.2444378
PLTP_HUMAN	0.7180146	0.0641231	0.2444378
PERL_HUMAN	0.5038272	0.0763513	0.2646845
FSP1_HUMAN	0.4001445	0.2080562	0.4626503
LV147_HUMAN	0.5034786	0.3074830	0.4996598
ZA2G_HUMAN	0.1295051	0.5520604	0.7176785
VP35L_HUMAN	0.2350438	0.6123376	0.7614320
IGKC_HUMAN	0.0340178	0.8323604	0.9017238
B2MG_HUMAN	0.0037347	0.9874597	0.9874597

## Data Availability

Not applicable.
